# Integrating Care for People With Co-Occurring Alcohol and Other Drug, Medical, and Mental Health Conditions

**Published:** 2011

**Authors:** Stacy Sterling, Felicia Chi, Agatha Hinman

**Keywords:** Alcohol and other drug use (AODU) disorders, comorbidity, co-occurring disorders, mental health, health care, treatment, treatment outcomes, integrated treatment, combined treatment

## Abstract

Most people with alcohol and other drug (AOD) use disorders suffer from co-occurring disorders (CODs), including mental health and medical problems, which complicate treatment and may contribute to poorer outcomes. However, care for the patients’ AOD, mental health, and medical problems primarily is provided in separate treatment systems, and integrated care addressing all of a patient’s CODs in a coordinated fashion is the exception in most settings. A variety of barriers impede further integration of care for patients with CODs. These include differences in education and training of providers in the different fields, organizational factors, existing financing mechanisms, and the stigma still often associated with AOD use disorders and CODs. However, many programs are recognizing the disadvantages of separate treatment systems and are attempting to increase integrative approaches. Although few studies have been done in this field, findings suggest that patients receiving integrated treatment may have improved outcomes. However, the optimal degree of integration to ensure that patients with all types and degrees of severity of CODs receive appropriate care still remains to be determined, and barriers to the implementation of integrative models, such as one proposed by the Institute of Medicine, remain.

It is widely recognized that the majority of patients with alcohol use problems also suffer from co-occurring mental health and medical problems. Co-occurring disorders (CODs) complicate the treatment process and, in many cases, contribute to poorer outcomes (Drake et al. 1996; Rosenthal and Westreich 1999) as well as higher service utilization and costs over time (Curran et al. 2008; Lennox et al. 1993). In the past, clinicians within each treatment setting—alcohol treatment, mental health, and general medicine—frequently treated COD patients as they would patients with only one of these disorders; however, such treatment is not well suited to the special needs of patients with CODs (Rosenthal and Westreich 1999). Extensive research has documented the need to treat all conditions from which patients suffer and has identified many key components of the best practices for achieving this goal (Goldman et al. 2000; Minkoff 1991; Minkoff and Ajilore 1998; Osher 1996). Moreover, a growing body of research suggests that integrated approaches to treatment may improve the outcomes of patients with alcohol problems (Craig et al. 2008; Drake et al. 2004, 2008; Goldman et al. 2000; Minkoff and Ajilore 1998; Osher 1996). Although optimally integrated care still is the exception in most treatment settings, interest in this approach is mounting, and many programs are attempting to incorporate integrated models of care.

This articles draws from the framework established in the Institute of Medicine (IOM) (2006) report, *Improving the Quality of Health Care for Mental and Substance-Use Conditions*, and other literature to consider the state of integrated care for people with alcohol problems and CODs. It examines how integrated approaches can make treatment more attractive to patients and contribute to higher retention rates and better outcomes, and discusses strategies and mechanisms that facilitate greater integration. It also considers barriers that impede optimal coordination of care for CODs, including organizational fragmentation; stigma; financing mechanisms; and the complex issues of confidentiality, patient safety, and the free flow of information necessary to implement integrated treatment approaches. Throughout the article, the term “disorder” refers to alcohol or other drug (AOD) use problems that meet the criteria set forth in the *Diagnostic and Statistical Manual of Mental Disorders, Fourth Edition* (DSM–IV) for abuse or dependence. The term “unhealthy use” describes less severe but problematical AOD use. The term “problems” encompasses the entire spectrum of severity.

## Scope of the Problem

### Prevalence of Co-Occurring AOD and Mental Health Problems

The high prevalence of co-occurring AOD problems and mental health conditions has been well documented in the addiction and psychiatric literatures. There are several excellent reviews of the epidemiologic research (Cornelius et al. 2003; Kessler 2004), and many studies of clinical samples (Compton et al. 2000; Flynn et al. 1996; Jainchill 1994; Sacks et al. 1997), as well as large national (Grant et al. 2004; Hasin et al. 2007; Kessler et al. 2005) and international (Kessler et al. 2007) population surveys, have been published. Lifetime prevalence of CODs among those seeking treatment for AOD disorders has been estimated at anywhere from one-quarter to well over one-half. For example, the National Comorbidity Survey, a general population survey of adults, found that 51.4 percent of those surveyed with a lifetime AOD disorder also reported a lifetime mental health disorder, whereas 50.9 percent of those with a mental health disorder reported having had an AOD disorder (Kessler 2004). The co-occurrence of AOD problems with mood and anxiety disorders is especially high. In a general population sample, the 2001–2002 National Epidemiologic Survey on Alcohol and Related Conditions (NESARC) found that of those with at least one AOD disorder, 20 percent suffered from a mood disorder and 18 percent from an anxiety disorder in the same period.

Many studies determine the prevalence of CODs by examining clinical DSM–IV diagnoses or by assessing patients’ scores on research instruments that are well validated and which typically assess type and severity of problems consistent with the criteria used to make DSM–IV diagnoses. The true prevalence of co-occurring AOD and mental health problems, however, probably is much higher than that documented in the literature, particularly when including lower-severity, subdiagnostic threshold cases. In addition, co-occurrence of AOD use and more than one mental disorder is not unusual (Jainchill 1994; Kessler et al. 2005).

### Chronology and Etiology of Co-Occurring AOD and Mental Health Problems

The chronology and etiology of CODs also are complex issues and often a contentious subject in the AOD treatment and psychiatry fields, because many of the factors that predispose patients to develop AOD use problems also are related to mental health problems. For example, on the one hand, AOD problems can stem from self-medication for mental health problems; on the other hand, they also can catalyze or exacerbate certain mental health problems (e.g., depression). The differences in how professional disciplines have perceived and addressed these complexities have contributed to the historical lack of treatment integration.

Regardless of the origin or order of problem development, however, the co-occurrence of AOD and mental health problems usually complicates the treatment process. In studies of treatment populations, psychiatric status has proven an important predictor of the course of AOD problems; in fact, it is one of the more salient and well-replicated variables associated with treatment seeking and lack of improvement (Haller et al. 1993; Hesselbrock 1991; McLellan et al. 1993; Rounsaville et al. 1987, 1991). In longitudinal population studies, psychiatric problem severity predicts increases in alcohol consumption and adverse consequences of drinking over time (Schutte et al. 1994). In addition to having poorer outcomes, AOD patients with psychiatric problems are at heightened risk of readmission (Booth et al. 1991; Moos et al. 1994*a*,*b*; Ornstein and Cherepon 1985).

### Prevalence of Co-Occurring AOD Problems and Medical Conditions

Co-occurring AOD problems and general medical conditions have been less studied than co-occurring AOD and mental health problems. However, the literature suggests that people with AOD problems have a higher prevalence of health problems in general and of many specific conditions in particular, including HIV disease, infection with hepatitis B and C viruses, hypertension, asthma, chronic obstructive pulmonary disorder (COPD), arthritis, headache, acid-related disorders, and many pain conditions (Cargiulo 2007; Carlsson et al. 2005; Corrao et al. 2000; Mertens et al. 2003). The AOD field has begun to develop a framework for examining the specific AOD abuse–related medical conditions that could be targeted for integrated interventions (Mertens et al. 2003). For example, COPD, depression, or hypertension patients could be targeted for alcohol screening (and brief treatment if appropriate) in primary-care or disease-management programs.

People with AOD disorders are at increased risk for many chronic medical conditions (Dickey et al. 2002; Mannelli and Pae 2007). As with mental health problems, clear etio-logic relationships are not easy to establish. Thus, unhealthy alcohol use is implicated in the development of some conditions (e.g., cirrhosis), increased exposure to some diseases (e.g., HIV, hepatitis), or exacerbation of existing medical problems (e.g., diabetes). Conversely, alcohol use also may result from attempting to cope with overwhelming medical problems (e.g., chronic pain). In addition, it is clear that medical conditions and their sequelae frequently interfere with the alcohol treatment process (e.g., doctor’s appointments may conflict with treatment program schedules or pain conditions may make it impossible to attend treatment) and impede recovery. Similarly, unhealthy AOD use can thwart medical treatments. For example, patients’ AOD use may impede their ability to comply with treatment regimens. In addition, AOD use is contraindicated with many medications and can inhibit immune system functioning.

## Integrating the Treatment of Co-Occurring AOD and Other Health Problems

### Co-Occurring AOD and Mental Health Problems

Although AOD treatment today occurs mainly in a separate system, it historically was located within the larger mental health treatment system. Until well into the 20th century, patients with alcohol problems—if they received treatment at all—received care from institutions and organizations charged with mental health care, such as asylums and sanatoria. (More often, alcohol problems were addressed within the criminal justice and, to a lesser extent, the social welfare systems.) The latter part of the 20th century saw the alcohol treatment field begin to separate from the mental health system in a variety of ways. Thus, programs were designed to specifically treat alcohol (and other drug) problems; the “disease model” of addictions and the attendant proliferation of the 12-step and self-help movements became more prominent, and research institutions dedicated to the formal study of AOD use problems, such as the National Institute on Alcohol Abuse and Alcoholism (NIAAA) and the National Institute of Drug Abuse (NIDA), were established. Many researchers and clinicians in the addictions field welcomed the separation because of concern that AOD problems had been given short shrift under the mental health system. The two separate public systems of care became largely funded by the Federal Government via separate block grants, further reinforcing the separation of services. Unfortunately, however, the separation also created a system in which most programs and providers do not have the resources, training, or inclination to treat patients with CODs and instead reinforced differences in provider attitudes toward specific disorders and in overall treatment philosophy. Regrettably, this often resulted in patients being referred to another agency for treatment of the other disorder before they were eligible to be seen for their presenting problem, or in ignoring the co-occurring problem entirely.

Differences between the mental health and AOD fields in clinician beliefs, training, behavior, and ideology pose significant barriers to the effective treatment of COD patients. On the mental health side it often has been argued that AOD problems are symptoms of deeper psychological distress and that when those other disorders are properly treated, AOD problems will lessen or subside. This conceptualization reinforces a hierarchy in which AOD disorders and their treatment are seen as less legitimate and less deserving of attention and resources. At the same time, the AOD treatment field frequently is ideology driven, and its disagreements with the mental health field on appropriate diagnosis and treatment often have been contentious.

Although AOD treatment programs may vary in other ways, the great majority have been influenced by the Alcoholics Anonymous (AA) tradition, and the major treatment model currently used in the United States, the “Minnesota Model” (IOM 1990; Kaskutas 1998; Room 1998), is based on the same 12-step principles. Although AA and AA-influenced programs have given much to the field (see below), they have had a pervasive unitary influence, resistant to competing treatment models (IOM 1990; NIAAA 1997), even in the case of CODs. These programs traditionally have emphasized more confrontational approaches than mental health programs, which have emphasized more supportive techniques (or have simply not treated patients until they are “clean and sober”). Many AOD treatment providers themselves are in recovery and graduates of AA and AA-influenced programs and adhere to a philosophy of abstinence. These treatment providers often frown on medications such as methadone or naltrexone for their patients, whereas medications are commonplace in mental health programs for psychiatric problems. This has significantly slowed the adoption of pharmacotherapeutic interventions for COD patients in many AOD treatment settings.

Screening and referral practices also differ. Historically, mental health providers have not routinely assessed patients for AOD misuse, and, by the same token, AOD treatment providers have not systematically screened for mental health problems. The reasons are many and in some cases may simply signify lack of training. However, too often assessment and diagnosis of CODs are ignored or delayed because the provider conceptualizes either the AOD or the mental health problem as “primary” and needing to be addressed before dealing with any other problems. Conversely, some clinicians may not feel equipped to treat patients with complex CODs, and prefer to refer them out to another agency for treatment. Both practices contribute to COD patients receiving suboptimal treatment.

Mental health and AOD treatment also have differed in their use of self-help groups. Whereas AOD treatment has a long tradition of relying on self-help, particularly 12-step–oriented groups, as a key therapeutic ingredient, they are much less commonly used in the psychiatric setting (Timko et al. 2005). Although the literature is mixed on whether COD patients are more or less likely than others to participate in 12-step meetings (Bogenschutz 2007; Chi et al. 2006*a*; Jordan et al. 2002; Kelly et al. 2003), evidence increasingly shows that when they do participate, they benefit from 12-step participation as much or more than other patients (Chi et al. 2006*a*; Magura et al. 2008; Timko and Sempel 2004). In the past two decades, self-help groups that are rooted in traditional 12-step programs but have been adapted to meet the special needs of people with CODs have been growing in number, and evaluations point to positive direct and indirect effects on several key components of recovery for COD patients (Magura 2008).

Clearly, reaching a consensus on treatment strategies that work for COD patients remains a challenge. However, this may be an opportune time to experiment with new treatment approaches. AOD treatment providers who see patients with CODs are becoming more open to trying new interventions (e.g., medications) for AOD disorders, as evidence for the effectiveness of these interventions is accumulating rapidly.

### Co-Occurring AOD Problems and Medical Conditions

Historically, alcohol and general medical services have been even less integrated than AOD treatment and psychiatry. Except for medically supervised detoxification, medical and AOD treatment providers continue to operate separately, although recent evidence suggests that integration would contribute to better outcomes (Friedmann et al. 2003; Grazier et al. 2003; Mertens et al. 2008; Weisner et al. 2001), and provide opportunities to intervene with patients who might benefit from AOD treatment (Aertgeerts et al. 2001; Bethell et al. 2001; Friedman et al. 1990; Singer et al. 1987).

For a variety of reasons—including discomfort with or insufficient knowledge about AOD problems, inadequate clinical tools, time constraints, ignorance of treatment resources, and issues of professional jurisdiction—many primary-care providers rarely screen for or discuss AOD use with their patients (Friedmann et al. 2000*b*; Spandorfer et al. 1999). Moreover, general medical practitioners only treat a small proportion of their patients’ AOD use problems.^1^ Stigma and societal attitudes about addictions affect physicians as well as the general public. Accordingly, many treatment providers are uncomfortable about discussing AOD use with their patients, and few are trained in assessment and treatment. The proliferation of “carve-outs”—arrangements whereby health plans contract with managed behavioral health care companies to provide AOD and mental health care services rather than reimbursing the providers—has reduced financial incentives for providers to treat patients rather than referring them (IOM 2006). As a result of all these factors, general medical practitioners are not commonly considered the appropriate health care professional to handle treatment for AOD use problems.

The role of general medicine in AOD treatment may be changing, however, because of increased interest in moving identification and brief treatment for AOD problems into medical settings in general, and primary care in particular. Evidence supporting the effectiveness of such interventions (Babor et al. 2005; Bertholet et al. 2005; D’Onofrio and Degutis 2002; Kanouse et al. 1995) is growing; moreover, several factors have been identified that can make such integrative practices more likely to succeed. These factors include the adoption of the drug and alcohol problem identification and treatment initiation measures set forth in the Healthcare Effectiveness Data and Information Set (HEDIS) of the National Committee for Quality Assurance (NCQA); the development of Current Procedural Technology (CPT) and Healthcare Common Procedure Coding System (HCPCS) codes that permit Medicare and Medicaid reimbursement for brief AOD treatments in medical settings; and NIAAA’s *Assessing Alcohol Problems: A Guide for Clinicians and Researchers, Second Edition* (2003) with accompanying evidence-based screening questions.

The growing evidence supporting the efficacy and effectiveness of medications for AOD problems also may encourage physicians to treat such problems, although studies suggest that pharmacotherapies for treatment of AOD disorders are adopted more slowly than for other medical conditions (Thomas et al. 2003). The extent of adoption of medications for AOD disorders also may be context related and depend on organizational policies and capacities (Fuller et al. 2005; Roman and Johnson 2002). For example, adoption of a new medication is more likely in settings where other AOD medications already are being prescribed (Knudsen et al. 2007); therefore, AOD medications are more likely to be adopted in AOD treatment programs than in primary care.

## Barriers to Integrating Care for Patients with CODs

AOD, mental health, and general medicine providers differ widely in education and training. Providers in medicine generally are physicians or advanced-practice nurses and mental health clinicians who typically hold doctoral- or master’s-level degrees. In contrast, the education and training among addiction treatment providers is more varied, ranging from medical or doctoral degrees to non-degreed peer counselors.

Organizational factors also pose significant barriers to the integration of care for patients with CODs. According to Ridgely and colleagues (1990, p.126), “The system problems are at least as intractable as the chronic illnesses themselves.” Most research indicates that people with CODs do not readily fit into either medical or traditional AOD treatment or psychiatry programs and that like patients with other chronic conditions they need ongoing services, possibly over several years (Mercer et al. 1998). This need for long-term services also is related to the issue of financing mechanisms for chronic-care patients (Tessler and Goldman 1992). On the whole, financing mechanisms currently are geared to acute rather than long-term treatment (Drake et al. 1996). Inclusion of reimbursement for long-term disease management of CODs might help lower hospitalization costs and improve outcomes. Related questions that should be addressed are whether treatment patterns and costs differ for different CODs and whether more coherent treatment policies could increase appropriate utilization of different treatment settings (i.e., primary care versus emergency departments versus inpatient care) and reduce costs.

Because of these complex organizational constraints, patients often are forced to navigate separate systems of care (sometimes both public and private), contacting different agencies or departments within large organizations (e.g., a health plan) and seeing multiple providers. Too often patients must coordinate their own care, even when appropriate linkages between providers and organizations are lacking. This can be especially challenging for patients experiencing cognitive and/or functional impairments related to their CODs, and, not surprisingly, many fail to follow through with one or more of their treatment regimens. Because of the stigma attached to co-occurring problems, many patients also experience considerable prejudice not only from society but from treatment providers, their own families, and even from themselves. Under these circumstances, it is difficult for patients to assume the role of proactive consumers, empowered to demand the highest quality, coordinated health care. As a result, many patients fall through the cracks in these fragmented systems of care, and treatment initiation, engagement and retention rates in this population are notoriously low (Chi et al. 2006*b*).

## Models of Treatment for Patients With CODs

Many programs now recognize the downside of separate systems for COD patients and are attempting to add integrative elements into their curricula. Currently, treatment models for patients with AOD problems and CODs broadly fall into four categories:

- *Serial treatment—*care is received in sequential treatment episodes, in separate systems of care;
- *Simultaneous/parallel*—care is received for both/all disorders simultaneously, but in separate, noncoordinated systems;
- *Coordinated/parallel*—care for both/all disorders is received simultaneously in separate but well-coordinated and closely linked systems, with established and formalized collaborative agreements; and
- *Integrated care*—care for both/all disorders is provided by the same cross-trained clinicians and in the same program, resulting in clinical integration of services.

Unfortunately, the evidence base for recommending one type or model of treatment over another is small. Controlled studies on integrated programs and services have been few, and the methodological challenges many, including small sample sizes (Ley et al. 2000). Moreover, most studies have focused on treatment for co-occurring AOD and mental health disorders, focusing particularly on patients with severe mental illness (Cleary et al. 2008; Drake et al. 2004; Dumaine 2003). A recent review of randomized clinical trials of psychosocial interventions to reduce AOD problems of severely mentally ill patients found no compelling evidence to recommend one type or model of treatment delivery over another (Cleary et al. 2008), partly because none of the models have been studied extensively (Cochrane 1999; Donald et al. 2005; Ley et al. 2008). The review by Ley and colleagues (2000) did not detect strong effects of different treatments on AOD outcomes. Only a few studies (Friedmann et al. 2003; Weisner et al. 2001) have examined the integration of *medical* care and AOD treatment.

Nevertheless, recent research has provided some evidence that integrated treatment may improve posttreatment outcomes (Drake et al. 2008; Godley et al. 1994; Meisler et al. 1997) or produce favorable outcomes compared with other types of services (Blankertz and Cnaan 1994; Drake et al. 1997; Herman et al. 2000) (also see the textbox). One study of AOD treatment patients with CODs (Grella and Stein 2006) found that patients in programs with more services for CODs (e.g., more “dual diagnosis” groups, higher percentages of clinicians with training or certification in COD treatment, or a higher number of psychological services) more frequently used psychological services and had better psychological and AOD use outcomes at 6 months. Another study (Craig et al. 2008) examined the impact on patient outcomes of training psychiatric clinicians in the treatment of CODs, including comprehensive assessment, motivational interviewing, and relapse prevention techniques. These investigators found that patients assigned to COD-trained clinicians had significantly better mental health outcomes at 18 months than did those who received usual mental health services. Other study findings have suggested that treatment components which increase integration of services for CODs may be beneficial. However, because many of these studies were of small samples, with most patients uninsured (often homeless) or on Medicaid, more research is needed “to compare outcome for non-homeless clinical patients in well-defined and monitored examples of integrated treatment and parallel treatment” (RachBeisel et al. 1999, p.1432).

### Fully Integrated Treatment: Is That the Goal?

In response to the growing evidence base for integrated care, one could argue that, ideally, all AOD treatment and mental health and medical programs should be fully clinically integrated—that is, all services should be provided simultaneously within the same organizations, by the same providers—and capable of treating patients with CODs. However, complete clinical integration does not seem feasible for most programs in the short term, if only for logistical reasons, particularly with regard to integrating medical care and AOD treatment. A recent survey estimates that only half of AOD programs nationwide offer dual AOD and mental health treatment (Mojtabai 2004), and even fewer offer integrated medical services. There is no evidence in the literature that mental health programs are more likely to coordinate services for patients with CODs. In fact, a survey of AOD and psychiatric treatment programs found that AOD programs were more likely to provide services for CODs than were psychiatric programs (Timko et al. 2005). Another strategy would be to incorporate specialty AOD and mental health services into general medical settings such as primary care. This approach could potentially reach far more patients in less stigmatized health care settings.

Another question is whether complete integration would even be desirable. For example, Minkoff (1997) suggested that full integration within programs actually might threaten choice, flexibility, and quality of treatment. Because COD patients are highly heterogeneous in their specific diagnoses and acuity, it is conceivable that integration and coordination of care across programs might be preferable to within-program clinical integration. History suggests that in fully integrated programs, patients with AOD and severe co-occurring mental health disorders are likely to receive the most attention, whereas patients with single disorders or with sub-diagnostic comorbidities are more likely to be excluded from treatment or their co-occurring problems not identified (IOM 2006).

**Impact of Integrated Care on Outcomes of Patients With Co-Occurring Disorders**

The findings of several Drug and Alcohol Research Team (DART) studies support prior research and clinical consensus that integrated care can improve outcomes for patients with co-occurring disorders (CODs):

- Alcohol and other drug (AOD) treatment patients with AOD abuse–related medical or psychiatric conditions who received integrated medical care and AOD treatment were more likely to be abstinent at 6 months than those who received usual independent medical care (69 percent vs. 55 percent; *P* < 0.006). The odds of total abstinence for the COD patients receiving integrated services was larger for the integrated than the independent treatment groups (odds ratio 1.90; *P* < 0.005) (Weisner et al. 2001). Receiving this integrated care during treatment continued to be related to remission for those with co-occurring conditions 5 years later (Mertens et al. 2008).
- Patients with co-occurring AOD and mental health conditions who received more hours of psychiatric services contemporaneously with their AOD treatment were more likely to report abstinence at 1 year (χ^2^ = 4.79, 1 df, *P* < 0.05). For those who had less than 2 months of concurrent COD and psychiatric services, the odds of being abstinent at 1 year were less than one-fourth of those with 2 and more months of services (χ^2^ = 7.94, 2 df, *P* < 0.05) (Chi et al. 2006*a*).
- Adolescent AOD treatment patients with co-occurring mental health disorders who received psychiatric services were more likely to be abstinent at 6 months than those who did not. Those who attended treatment in AOD programs that were colocated with mental health clinics had higher odds of abstinence from both alcohol and drugs (odds ratio 1.57 [95% confidence interval: 1.03–2.39]), drugs (1.84 [1.87–2.85]), and of returning after intake to initiate COD treatment than others (2.28 [1.44–3.61]; *P* < 0.001) (Sterling and Weisner 2005).

Although the evidence does not point to a single optimal level of integration, accrediting bodies, purchasers, and Federal and State agencies can greatly facilitate integration of services by implementing certain overarching strategies, identified by the IOM Committee (see the table). The IOM (2006) report endorses a conceptual model that was developed by Friedmann and colleagues (2000*a*) (see the figure) to illustrate the spectrum of care integration. In this model, according to Friedmann and colleagues (2000*a*, p. 445), mechanisms for coordinating services range from “the ad hoc, market-based purchase of services from local providers to the complete control and coordination of a fully integrated, centralized service delivery system.” It seems entirely plausible that more extensive and formalized integrative mechanisms would improve the quality of care for patients with CODs and would offer the best chance of improving their outcomes. It is worth noting however, that this model emerged from an examination of how service coordination affected service utilization of drug treatment patients; it did not specifically address services for CODs, and did not examine patient outcomes beyond utilization. Thus, much more research needs to be conducted comparing the organization of care for CODs.

The flow of confidential information poses a complex barrier to implementing integrated care for patients with CODs. Patient health information is carefully (and rightly) protected, and information about the treatment of AOD problems is particularly well-guarded by Federal and State regulations and organizational policies, such as 42 CFR, part 2 (Electronic Code of Federal Regulations (e-CFR) 2009). Although preventing sensitive and potentially damaging patient information from falling into the wrong hands is essential, these regulations, originally designed to protect drug-treatment patients from legal prosecution, have had the unintended consequence of inhibiting the coordination of health care across agencies and departments. The stringent requirements for obtaining consent to release information (especially challenging for some patients with CODs) may inhibit coordination of care, enhanced referral, consultation, and follow-up. For example, integration of care may be compromised if a provider in one program cannot determine if a patient has followed through with a referral, or if a patient has a health condition that is related to, could be exacerbated by, or requires medication which is contraindicated with AOD use. Moreover, these regulations and practices can serve to reinforce the stigma associated with AOD and mental health problems.

The IOM recommends that sharing of information between providers treating the same patient become more routine. Clinicians should discuss with each patient the importance of sharing diagnoses, medications, and other therapies between providers treating CODs to enable collaborative care between clinicians. The report acknowledges that information on mental health and AOD conditions is sensitive and that sharing this information often is governed by Federal and State laws and individual organization practices. The report therefore calls on State and Federal entities and organizations implementing additional information policies to re-examine their policies and practices on information sharing to ensure that they are not inappropriately interfering with coordinating care (IOM 2006). The rapid development of health information technology (IT) and the growing adoption of electronic medical records further complicate these issues. Integrated health IT systems could potentially contribute significantly to the integration of care for patients with CODs and improve the quality of care, and the field must carefully weigh these potential benefits against privacy concerns. Several leading policy groups are considering this issue, which was included as one of the key strategic areas at the “National Summit on Defining a Strategy for Behavioral Health Information Management and Its Role Within the Nationwide Health Information Infrastructure,” convened in 2005 by the Substance Abuse and Mental Health Services Administration (SAMHSA). The summit concluded that “Legal issues should be clarified and in some cases changed to facilitate appropriate information sharing across service systems for care coordination and service improvement” (Substance Abuse and Mental Health Services Administration and Software and Technology Vendors’ Association 2005, p. 2).

## Discussion

Many factors have converged to focus attention on the nature and quality of health care for people with CODs, not least of which is the realization that, whatever the causes, these patients have not been served well by the traditional treatment system(s). As a result, there seems to be a greater openness to considering new models of care for these patients. For example, in 2009 NIAAA, NIDA, and the National Institute on Mental Health (NIMH) came together at a conference entitled “Integrating Services, Integrating Research for Co-Occurring Conditions: A Need for New Views and Action” to begin to focus on a new agenda for collaborative research on CODs. Other developments, such as the adoption of HEDIS performance measures discussed above and the enactment of national mental health and addiction treatment parity legislation, surely will have an impact on the integration of services for CODs. Furthermore, the rapid evolution of health IT systems will undoubtedly shape the way patient information is shared between programs and providers and has the potential to increase collaboration significantly if concerns about patient privacy are adequately addressed. All of these environmental developments merit close observation and study as they evolve.

Clearly, changes in the health care system and in models of service delivery also will affect the way care is organized for all patients, not only those with CODs. Advocates of a model called patient-centered medical home (PCMH)^2^ have called for including behavioral health services in a fully integrated model for delivering primary care, AOD, and mental health services (Arvantes 2008; Croghan and Brown 2010), consistent with the current health care reform discussions that stress less fragmentation in service delivery (Rittenhouse and Shortell 2009). A broad coalition of health care stakeholders, including 17 specialty societies (e.g., the American College of Physicians, the American Academy of Pediatrics, and the American Academy of Family Physicians), have endorsed the model, and it currently is being tested through demonstration pilot projects in some major public and private health plans (Berenson et al. 2008; Rittenhouse and Shortell 2009). A full understanding of this model and its strengths and limitations is still evolving (Berenson et al. 2008; Sidorov 2008), but it likely would increase coordination and quality of care for patients with CODs.

As previously noted, integrated treatment for CODs has not been studied extensively, and the field needs to compare different interventions and combinations of interventions, preferably in carefully controlled trials. Because of the sparse research, it is especially important to study models of care integrating medical and AOD treatment (such as the PCMH mentioned above), whether in medical settings or in AOD programs. Because most research and program development have focused on patients with co-occurring severe AOD disorders and severe mental illness, it also is necessary to examine the effects of integrated treatment interventions and models on patients with lower-severity CODs, including those who may not meet diagnostic criteria for specific disorders (e.g., DSM diagnoses for depression, anxiety, AOD abuse or dependence) but whose co-occurring problems impede their chances for positive outcomes. These patients comprise a much larger group than those with severe CODs but may be underserved in programs where patients with more severe conditions receive more clinical or program attention. Thus, policymakers and program planners seeking to improve health care systems for COD patients must take care to not “integrate” programs to an extent that non-COD patients, especially those with AOD problems, effectively are excluded from treatment because they do not meet diagnostic criteria. Models of services delivery such as the “quadrant model” of care, which has been endorsed by the National Association of State Alcohol and Drug Abuse Directors, should be considered and incorporated. This model, which emphasizes a continuum of chemical dependency and mental health services based on the combined severity of co-occurring AOD and mental health problems, explicitly includes lower-severity patients whose treatment might take place in any of the three treatment contexts (i.e., AOD, mental health, or medical settings) (IOM 2006).

Beyond studying specific interventions, however, it is necessary to evaluate programs’ and systems’ overall COD competency. Researchers and policymakers have argued that broader best practices need to be developed that “apply to the entire system of care and that require integrated system planning involving both MH and SA treatment agencies,” and that “… a focus on best practices at the program level is being replaced by a focus on the system level.”(Minkoff 2001, p. 597) This systems-level research should include studies of the develop ment, refinement, and dissemination of measures of organizational COD capacity (McGovern et al. 2007).

Advocates for change have influenced providers and policymakers who serve patients with CODs. It now is generally acknowledged that these patients have had to navigate fragmented systems and that they have received treatment that is less accessible and less effective than the health care system has the potential to deliver. After years of underestimating the presence of CODs, providers and policymakers now recognize that these conditions are highly prevalent and that, in fact, the majority of patients with AOD problems most likely have a COD. Research on the effectiveness of interventions and models of care for treating CODs has substantially grown in recent years and now is a major focus of the leading research institutes. This is an exciting time for the field. Although the challenges of providing (and studying) integrated services for patients with CODs remain, health care stake holders are accumulating the research and building the organizational models to support substantial advances in providing more easily accessible treatment with the potential to greatly improve outcomes for patients with CODs.

## Figures and Tables

**Figure:**
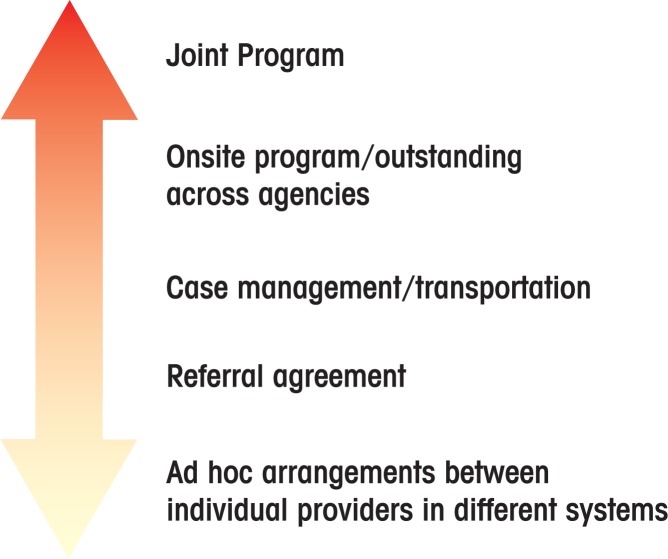
Continuum of care coordination for patients with alcohol and other drug use disorders and co-occurring disorders ranging from mild severity (bottom) to high severity (top). SOURCE: Friedmann et al. 2000*a*.

**Table:** Institute of Medicine (IOM) Recommendations for Implementing Quality Integrated Care for Individuals With Co-Occurring Disorders (CODs).

• Coordination of care and integrated treatment by leadership and all key stakeholders. Development of a shared vision among systems of care (Minkoff 1991, 1997, 2001; Mueser et al. 2003).
• A “no wrong door” policy. Wherever individuals enter a service system, they will find access to care, including “anticipation of comorbidity and formal determination of intent to treat or refer.”
• Clear and agreed-upon definitions of coordination of care, formally documented between providers and in purchaser agreements. This will help ensure coordination and accountability for outcomes.
• Assertive outreach and patient engagement and retention activities, key to improving outcomes for COD patients.
• Development and adoption of standardized performance indicators across organizations and systems.
• Comprehensive assessment practices across systems of care (e.g., alcohol and other drug treatment programs, mental health departments, primary care, chronic-disease programs, and emergency departments). The IOM specifically recommends (1) screening for alcohol misuse by all adults, including pregnant women (U.S. Preventive Services Task Force); (2) screening for a co-occurring mental or substance-use problem at initial presentation with either condition; and (3) screening of entrants into child welfare and juvenile justice systems, because of the high prevalence of CODs among children (IOM 2006). Assessments on-site when possible, by referral when necessary.
• Interdisciplinary training of staff, to enhance clinical capacity and fluency with diagnostic and treatment placement criteria, and therapeutic techniques, regardless of type of program.
• Comprehensive services across programs and across disorders (e.g., individual and group therapy, family therapy, vocational counseling, assistance with housing and income programs, case managements, etc.).
• All types of disorders treated as “primary.” No program, patient, type of disorder, or approach to treatment is considered more important than others.
• Motivational enhancement activities, which studies show are among the most effective components of care (Cleary et al. 2008).
• Availability of long-term services and continuity of care across programs and time. Patients may benefit from a disease management/chronic care rather than an episodic treatment approach.
• “Reduction of negative consequences” or harm-reduction philosophy (Mueser et al. 2003). Improvement in mental health symptoms and functioning should be emphasized as important interim goals.
• Compatible administrative infrastructures, including information technology systems and instruments, electronic medical records, and assessment tools.
• Sharing of patient information, including patient records when possible, and encouragement of patients to consent to releasing information. Programs should require clear guidelines and safeguards around the use, disclosure, and protection of confidential health information.
• Flexible funding across systems to reduce barriers posed by distinct financing mechanisms.
• Colocation of services and clinicians whenever possible (Friedmann et al. 2000*a* ; Hellerstein et al. 1995; Sterling and Weisner 2005).
• Clinical integration of services whenever possible (i.e., dual services provided by the same clinicians, or clinicians in the same programs).
• Program and organizational linkages with other systems involved with the patient (e.g., criminal justice and welfare systems, schools, and employee assistance programs).

## Footnotes

1 However, treatment may occur more often than reported, because physicians may code their patients’ alcohol disorders as somatic complaints for which they can be reimbursed.

2 The PCMH is a model of a primary care organization that delivers the core functions of primary health care in a manner that is patient-centered, comprehensive, coordinated, allows better access, and emphasizes quality and safety improvement) (Agency for Healthcare Research and Quality 2010).
